# Automated mass spectrometry‐based profiling of multi‐glycosylated glycosyl inositol phospho ceramides (GIPC) reveals specific series GIPC rearrangements during barley grain development and heat stress response

**DOI:** 10.1111/tpj.70279

**Published:** 2025-06-26

**Authors:** Marlene Pühringer, Nina Thür, Madeleine Schnurer, Leonida M. Lamp, Lisa Panzenboeck, Jürgen Hartler, Andrea Tanzer, Verena Ibl, Evelyn Rampler

**Affiliations:** ^1^ Department of Analytical Chemistry, Faculty of Chemistry University of Vienna Währinger Str. 38 Vienna 1090 Austria; ^2^ Vienna Doctoral School in Chemistry (DoSChem) University of Vienna Währinger Str. 42 Vienna 1090 Austria; ^3^ Department of Functional and Evolutionary Ecology, Molecular Systems Biology (MoSys) University of Vienna Djerassiplatz 1 Vienna 1030 Austria; ^4^ Vienna Doctoral School of Ecology and Evolution (VDSEE) University of Vienna Djerassiplatz 1 Vienna 1030 Austria; ^5^ Institute of Pharmaceutical Sciences University of Graz Universitätsplatz 1 Graz 8010 Austria; ^6^ Field of Excellence BioHealth University of Graz Humboldtstraße 50 Graz 8010 Austria; ^7^ Research Network Data Science University of Vienna Kolingasse 14‐16 Vienna 1090 Austria; ^8^ Faculty of Agriculture, Environment, Chemistry University of Applied Sciences Dresden Pillnitzer Platz 2 Dresden Germany

**Keywords:** glycosyl inositol phospho ceramides, GIPC, barley, plant membrane, heat stress, glycosphingolipid, mass spectrometry, liquid chromatography, fragmentation, automated annotation

## Abstract

Glycosyl inositol phospho ceramides (GIPC) are the predominant glycosphingolipids in plant membranes, essential for their membrane stability, cell signaling, stress adaptation, and pathogen resistance. However, their complex structures, characterized by a ceramide backbone and a glycan head group, have challenged comprehensive analysis using traditional methods, which often rely on separate glycan or lipid profiling. To overcome these limitations, we developed a glycosphingolipidomics assay using reversed‐phase high‐resolution mass spectrometry including multistage fragmentation (RP‐HRMS^n^). This method enables direct, detailed structural characterization of GIPC in plants, combining advanced chromatographic separation, multistage fragmentation, and automated annotation using decision rule‐based criteria. Applied to barley grains, the assay identified 102 GIPC species, including A‐, B‐, C‐, and D‐series GIPC, previously unreported glycan branching fragments (421 and 403 *m/z*), and a huge structural variety in the ceramide moiety. Profiling at different development stages revealed dynamic GIPC regulation during grain development, with an upregulation of B‐ and C‐series towards mature development stages. The application of heat stress induced significant remodeling of GIPC profiles, mainly through upregulation of B‐series species, which emphasizes their roles in maintaining membrane stability and functionality under abiotic stress conditions. The presented glycosphingolipidomics assay enables the first automated analysis of complex GIPC through a decision rule‐based identification approach. By resolving GIPC to the molecular lipid species level, the method provides novel insights into GIPC diversity, homeostasis, and their critical roles in membrane dynamics, stress adaptation, and pathogen resistance, paving the way for advanced research in plant lipidomics and stress biology.

## Significance Statement

This study introduces an automated method for detailed profiling of complex glycosyl inositol phosphor ceramides (GIPC), revealing their structural diversity and dynamic roles in barley grain development and stress responses. By advancing our understanding of GIPC in membrane stability and stress adaptation, this work provides a valuable tool to explore plant lipidomics and resilience mechanisms, with implications for crop improvement under environmental challenges. Created in https://BioRender.com.

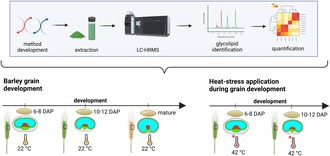

## INTRODUCTION

**Figure 1:**
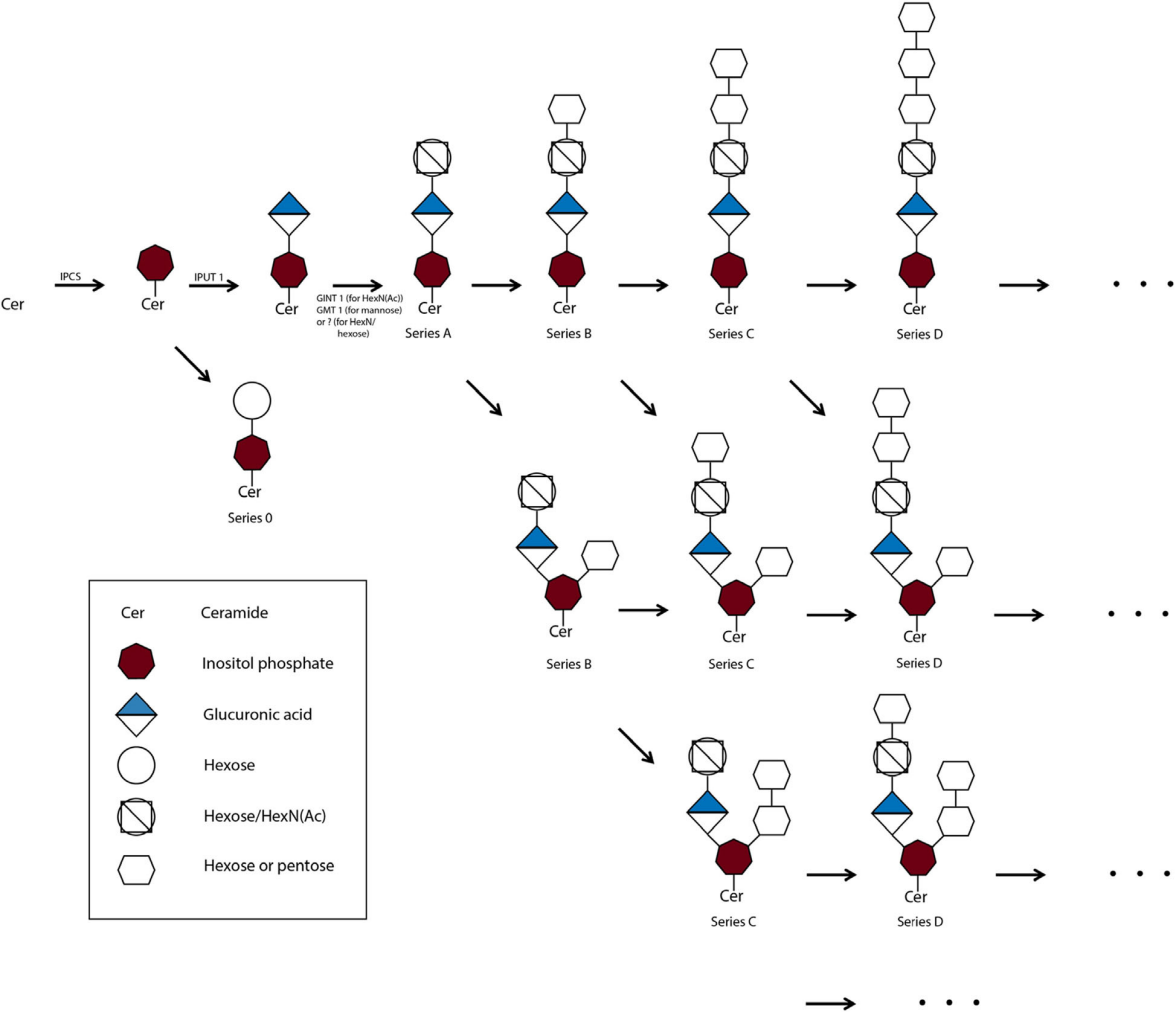
Glycan structures of GIPC series A–F demonstrate the progressive increase in length across the series, with glycan branching possible at the inositol phosphate in higher series (B and beyond). GMT 1, glycosylinositol phosphorylceramide mannosyl transferase 1; GINT 1, glucosamine inositol phosphorylceramide transferase 1; IPCS, inositol phosphorylceramide synthase; IPUT 1, inositol phosphorylceramide glucuronosyltransferase 1.

Glycosyl inositol phospho ceramides (GIPC) are the predominant sphingolipids in earth's total biomass, as they are an integral part of the membranes of plants and algae (Gronnier et al., 2016). For example, GIPC comprise approximately 64% of total sphingolipids and 25% of the plasma membrane lipids in the leaf of the dicotyledon plant *Arabidopsis thaliana* (*A. thaliana*) (Buré et al., 2011; Cacas et al., 2013, 2016; Markham et al., 2006; Markham & Jaworski, 2007). GIPC have been suggested to play a role in (i) organizing the plasma membrane by contributing to the lipid bilayer asymmetry and the formation of lipid rafts (Cacas et al., 2016); (ii) cell signaling by binding, for example, Na^+^, thereby triggering Ca^2+^ influx in response to salt stress (Jiang et al., 2019), and through the production of signaling molecules via hydrolysis of GIPC by GIPC‐specific phospholipase D (Hasi et al., 2019; Tanaka et al., 2013); (iii) cell wall anchoring by interacting with rhamnogalacturonan II (R‐II) via Boron, responsible for the interaction between the plasma membrane and cell wall (Begum & Fry, 2023; Funakawa & Kyoko, 2015; Voxeur & Fry, 2014); (iv) plant immunity, enhancing sensitivity to rice blast disease (Lin et al., 2023); (v) plant growth and development, affecting, for example, pollen fertility (Lin et al., 2023; Rennie et al., 2014; Tartaglio et al., 2016); (vi) facilitating homeoviscosity (Mamode Cassim et al., 2021). GIPC were first structurally characterized in 1958 (Carter et al., 1958) and are composed of a ceramide backbone linked to an inositol glucuronic acid unit via a phosphodiester bond (Buré et al., 2014; Gronnier et al., 2016; Ishikawa et al., 2016). In plants, up to 19 saccharides including arabinose, galactose, mannose, and fucose can be linked to the phospho inositol substructure (Buré et al., 2014; Kaul & Lester, 1975; Pata et al., 2010). Like in other plant sphingolipids, phytosphingosine is used as a sphingolipid precursor to form an amide bond with a fatty acid (FA), resulting in the ceramide core structure of GIPC. In plants, yeast, and protozoans, the condensation of ceramide with inositol phosphate is mediated by inositol phosphorylceramide synthases (IPCSs) in the Golgi (Mortimer & Scheller, 2020; Nagiec et al., 1997; Wang et al., 2008). The resulting inositol phospho ceramide (IPC) is then modified within the Golgi by the addition of glucuronic acid (GlucA) catalyzed by inositol phosphorylceramide glucuronosyltransferase 1 (IPUT1) to produce GlucA‐IPC. The gene encoding IPUT1 was first identified in *A. thaliana* (Rennie et al., 2014). In *A. thaliana*, GlucA‐IPC is a precursor of mannose‐GlucA‐IPC, where a mannose residue is added by the glycosylinositol phosphorylceramide mannosyl transferase 1 (GMT1) to finally produce an A‐series GIPC (Fougère et al., 2024). The attached sugar present in A‐series and higher series GIPC is highly tissue‐specific and can be either mannose, glucose, *N*‐acetylglucosamine (GlcNAc) or glucosamine (GlcN) (Haslam & Feussner, 2022). While GMT1 mediates mannose attachment, glucosamine inositol phosphorylceramide transferase 1 (GINT1) adds GlcNAc and GlcN to the GlucA‐IPC backbone (Ishikawa et al., 2018). Analysis of GIPC in *A. thaliana* and *Nicotiana tabacum* revealed six GIPC (A–F) series. The series identifier A–F represents the number of saccharide units: starting with A‐series GIPC, which comprise two saccharide units, each subsequent letter stands for an additional saccharide, resulting in seven units for the F‐series (Buré et al., 2011) (Figure 1). An overview of GIPC glycan synthesis, branching, and known involved enzymes is provided in Figure 1. Systematic nomenclature of GIPC specify the number of glycans (series A–F), the type of glycans and rest groups (e.g., Hex/Pen; NH/NAc), and the number of carbons, double bonds, and hydroxylations in the ceramide moiety (e.g., 18:1;O3/20:0;O).

**Figure 2:**
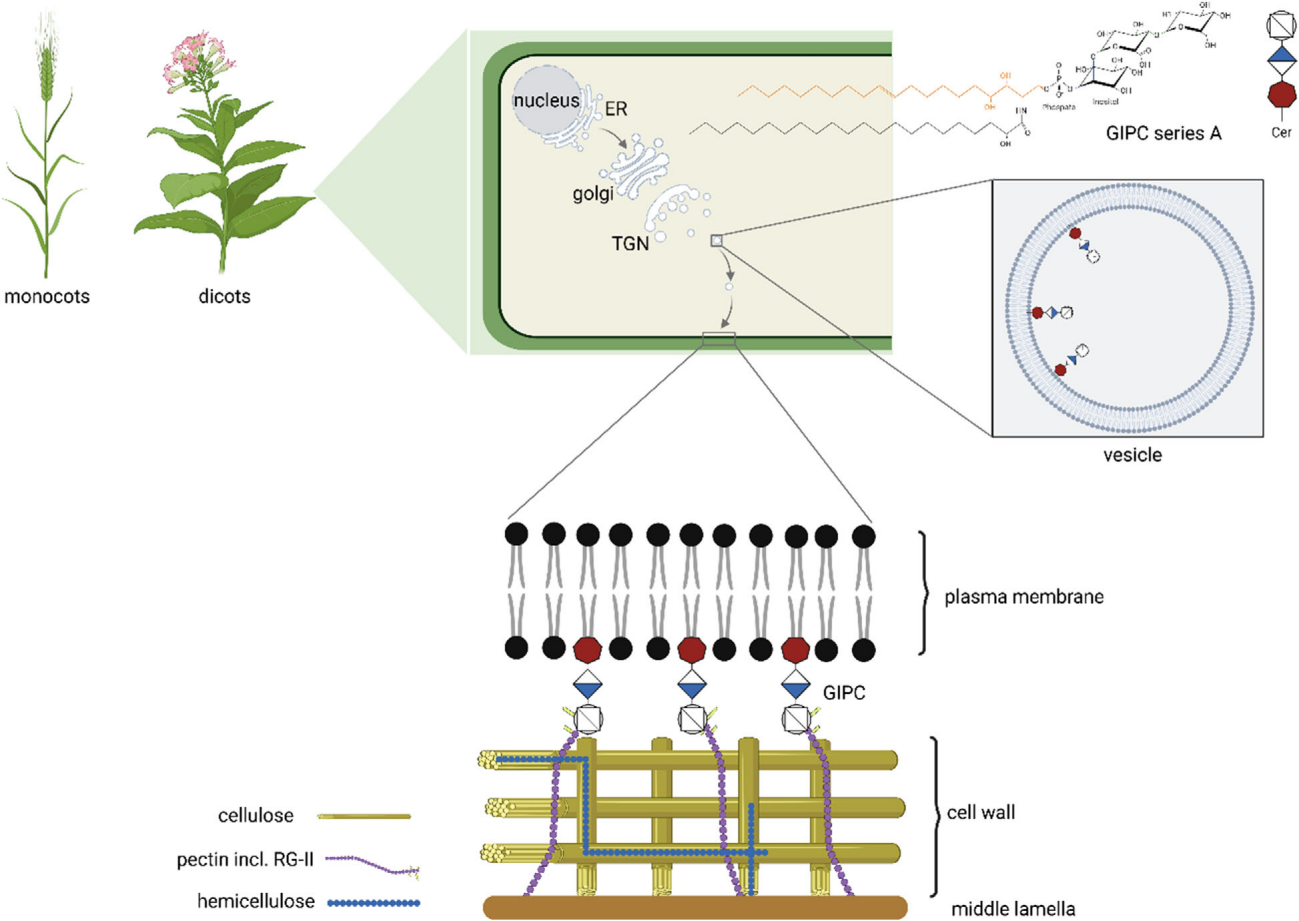
Transport and localization of GIPC in plant cells. A‐series GIPC, chosen as the simplest form, is used as an example to illustrate the glycan head group and ceramide backbone structure characteristic of all plant GIPC. GIPC are synthesized in the ER, modified in the Golgi, and transported via vesicles to the outer plasma membrane. As an example, for GIPC function the interaction of GIPC with RG‐II is depicted, which ensures that the plasma membrane is tightly linked to the cell wall. Created in https://BioRender.com.

The synthesized GIPC are subsequently transported to their final destination in the plasma membrane (PM) (Figure 2). Notably, GIPC series are species‐ and tissue‐specific and vary between monocots and dicots: Whereas A‐series GIPC are most abundant in dicots as in *A. thaliana*, B‐series GIPC are predominantly abundant in monocots as in *Zea mays* (Cacas et al., 2013; Mamode Cassim et al., 2021). Within dicots, F‐series GIPC were only identified in tobacco BY‐2 cell culture (Buré et al., 2011). Thus, it can be assumed that the function of GIPC is species‐dependent.

The identification and quantification of GIPC is still an ongoing challenge. Since GIPC has both a sugar head group and a lipid subunit, resulting in extremely complex glycosphingolipid combinations, specialized extraction methods are required to handle the amphiphilic properties of GIPC and the characteristics of the plant material. Accordingly, tailored analytical detection methods are required for annotating these complex glycosphingolipids. To address this methodological gap, we developed a method for automated profiling of GIPC in plants using the combination of liquid chromatography (LC) and mass spectrometry (MS). LC–MS‐based methods allow for the parallel monitoring of hundreds of native (glyco‐)sphingolipids, which can be identified based on their retention time (RT), mass‐to‐charge ratio (*m/z*), and fragmentation pattern (Buré et al., 2014; Hohenwallner et al., 2022, 2024; Tarazona et al., 2015). In this study, multistage fragmentation (MS^n^) followed by automated annotation was applied to elucidate the complex structure of glycosphingolipids. This workflow enabled the confirmation of substructures within the sugar unit of A–F series GIPC through additional MS fragments or determination of the number of hydroxylations on the two carbon chains.

Since GIPC profiles are affected by abiotic stress (cold‐ and drought‐induced membrane remodeling) (Tarazona et al., 2015) and are more abundant in barley roots after salt treatment (Yu et al., 2018), we chose barley grains as a monocot model system to apply our newly developed method. Subsequently, in this study, we applied our new method to identify a GIPC profile specific to barley grains during grain development in natural conditions and after heat stress application at different development stages. It is known that different tissues within the barley grain undergo physiological rearrangements during grain development under natural conditions, including programmed cell death (PCD), and grain moisture down to almost complete dryness (0%) in the mature stage (Alvarez Prado et al., 2013). In parallel, drought and heat stress induce GIPC accumulation by increasing the sugar content of membrane head groups (Tarazona et al., 2015). The increased sugar content likely prevents protein precipitation during desiccation and serves as a reservoir for the enhanced synthesis of phosphatidyl inositol (Gasulla et al., 2013). Our study demonstrates the successful application of our new method, targeting specifically GIPC, revealing B‐series GIPC specific for development and heat stress response in young barley grains.

## RESULTS

In a previous study, we developed the automatic identification of A‐series GIPC showcased in various plants (Panzenboeck et al., 2020). To further enhance this method, we set up two experiments: first, we analyzed the GIPC rearrangement within developing barley grains, without temperature stress application. We chose barley as a model, as it is an economically important crop and a model for cereal genetics and genomics (Sreenivasulu et al., 2008, 2010). It is known that the grain undergoes a significant moisture reduction, particularly from the grain‐filling stage to maturity (Alvarez Prado et al., 2013). This water loss increases the membrane permeability and the cell wall‐membrane interaction due to structural changes, which lead to differences in elasticity (Feng et al., 2016) and therefore might go along with GIPC profile changes. Second, we applied heat stress at two different early stages of barley grain development. Since it is known that GIPC production is induced by heat stress, we analyzed the GIPC rearrangement after heat stress application. In both experiments, we applied our newly developed method to identify and quantify relative changes of complex GIPC. Finally, both GIPC profiles were compared and present specific GIPC during barley grain development and after heat stress application.

### GIPC profiling based on RP‐HRMS^n^ in barley grains uncovers fragments designating glycan branching

**Figure 3:**
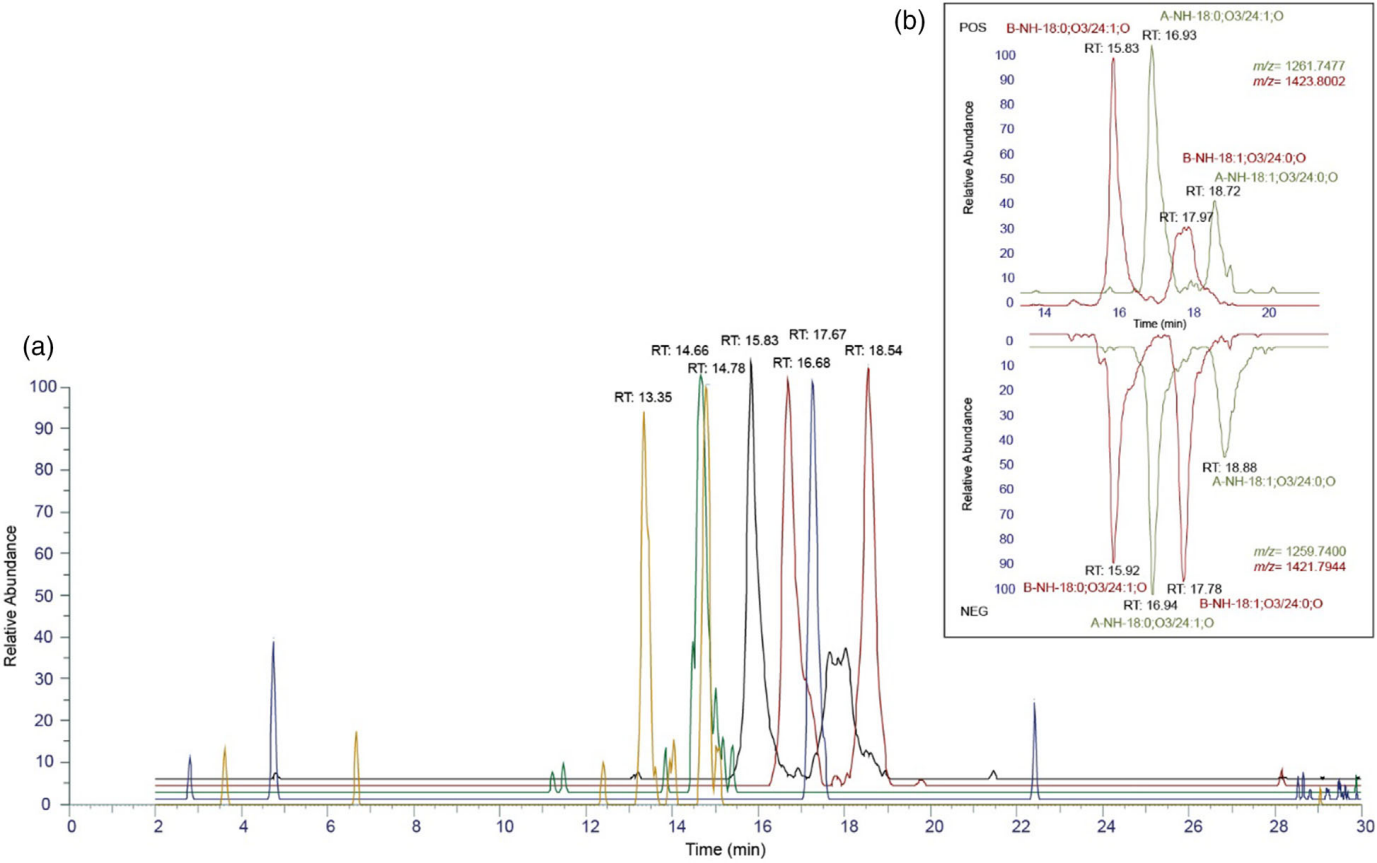
(a) Extracted ion chromatograms of five abundant GIPC species identified in barley samples: B‐NH‐40:3;O3 at RT = 13.35 min & 14.66 min (yellow), A‐NH‐18:0;O2/18:0 at RT = 14.66 (green), B‐NH‐18:0;O3/24:1;O at RT = 15.83 min (black), B‐NH‐18:1;O3/24:0;O at RT = 17.94 min (black), B‐NH‐18:0;O3/24:1 at RT = 16.68 min (red), B‐NH‐18:1;O3/24:0 at RT = 18.54 min (red), B‐NAC‐18:0;O3/24:0;O at RT = 17.67 min. (b) Extracted ion chromatogram of the isomers B‐NH‐18:0;O3/24:1 and B‐NH‐18:1;O3/24:0, as well as A‐NH‐18:0;O3/24:1 and A‐NH‐18:1;O3/24:0 in positive ion mode (top) and negative ion mode (bottom).

We developed a glycosphingolipidomics workflow combining reversed‐phase (RP) chromatography, high‐resolution mass spectrometry (HRMS), and multistage fragmentation (MS^n^) to profile GIPC in barley grains. Sample preparation involved homogenizing barley grains, adding a chemically similar internal standard, inhibiting enzymatic and oxidative degradation, and performing one‐phase extraction with alkaline hydrolysis. For GIPC profiling, we employed an RP‐MS^n^ method with a GIPC inclusion list of theoretical masses, MS2/MS3 fragmentation in positive and negative ion modes, and product ion triggers for fragments at 259 and 241 *m/z* (inositol phosphate and its water loss). Structural characterization was based on *m/z* information, retention time matching of positive and negative ion mode (Figure 3), diagnostic fragments, and the equivalent carbon number model (Ovčačíková et al., 2016; Panzenboeck et al., 2020).

**Figure 4:**
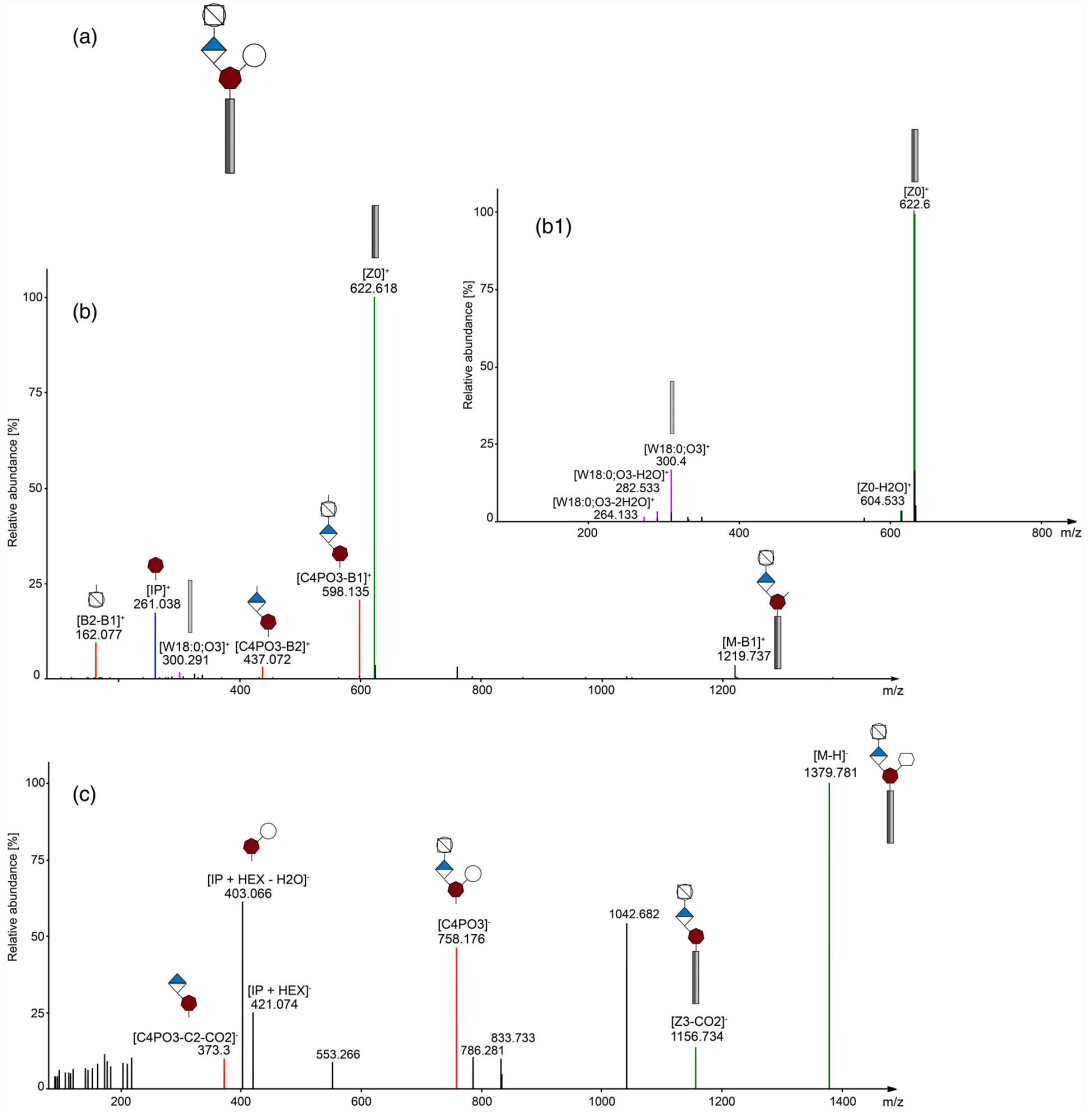
MS^n^ spectra of GIPC B‐NH‐18:0;O3/22:0 identified in barley samples. (a) Chemical structure of B‐NH‐18:0;O3/22:0. MS^n^ peaks are assigned to their fragments depicted in symbol nomenclature where the gray rectangles represent the fatty acid and sphingoid base while the sugar part is depicted as introduced in Figure 1. Fragments are named with letters (B, C, Y, Z, V, W) as introduced by Domon and Costello (1988). (b) MS2 spectrum of the [M + H]^+^ precursor ion in positive ion mode. (b1) MS3 spectrum of Z0^+^ fragment (622 *m/z*) in positive ion mode. (c) Negative ion mode MS2 spectrum of the [M‐H]^1−^ precursor ion. The IP + HEX‐H_2_O and IP + HEX fragments are indicative for branched GIPC structures and were detected in negative ion mode.

Automated GIPC annotation was performed based on a set of in‐house developed decision rules for the freely available software LDA (Hartler et al., 2017). An in‐depth structural analysis by fragmentation pattern was possible using the developed RP‐HRMS^n^ method. A‐series GIPC (Hex(R1)‐HexA‐IPC) consistently produced characteristic fragments for the glycan (B and C fragments) and lipid moieties (Y, Z, and V, W fragments) (Domon & Costello, 1988). Characteristic fragments for GIPC are inositol phosphate (IP) and its water loss (IP‐H_2_O), which can be observed in positive ion mode. Figure 4 shows mass spectra of GIPC B‐NH‐18:0;O3/22:0 in both ion modes as well as an MS3 spectrum in positive ion mode. Fragments that, to the best of our knowledge, are indicative of branched structures could be annotated in MS2 spectra of [M‐H]^−^. The fragments at 421 *m/z* and 403 *m/z* correspond to the masses of IP + hexose (IP + HEX) and IP + hexose‐H_2_O (IP + HEX‐H_2_O) respectively, which is a sign of the last glycan unit (hexose) being attached to the inositol and thereby creating a branched GIPC structure. Additionally, a GIPC branching fragment (535 *m/z*) has already been described by Buré et al. (2016) and was also observed in C‐series spectra of the barley samples.

Using multistage fragmentation, we characterized the lipid composition of the sphingoid base (SPB) and the fatty acid (FA) chain to resolve the molecular species level of GIPC. Our analysis identified GIPC across series A–D with 2–4 hydroxylations. The SPBs consistently comprised 18 carbons in barley and were either dihydroxylated or trihydroxylated (18:0;O2, 18:1;O2, 18:0;O3 or 18:1;O3). Among these, 18:0;O3 was the most abundant SPB, with 18:1;O3 also showing high abundance. FA chains ranged between 16 and 26 chain length, with even and odd carbon numbers, and were non‐ or singly hydroxylated. The highest amount in terms of normalized ratio to gram dry weight were FA 24:0 and FA 24:0;O. All GIPC were either identified on the species level (16 species), given as a sum of fatty acid and SPB (e.g., 42:0;O3), or molecular species level (86 molecular species), in which specific FAs and SPBs can be assigned (e.g., 18:0/24:0;O3) (Liebisch et al., 2020).

**Figure 5:**
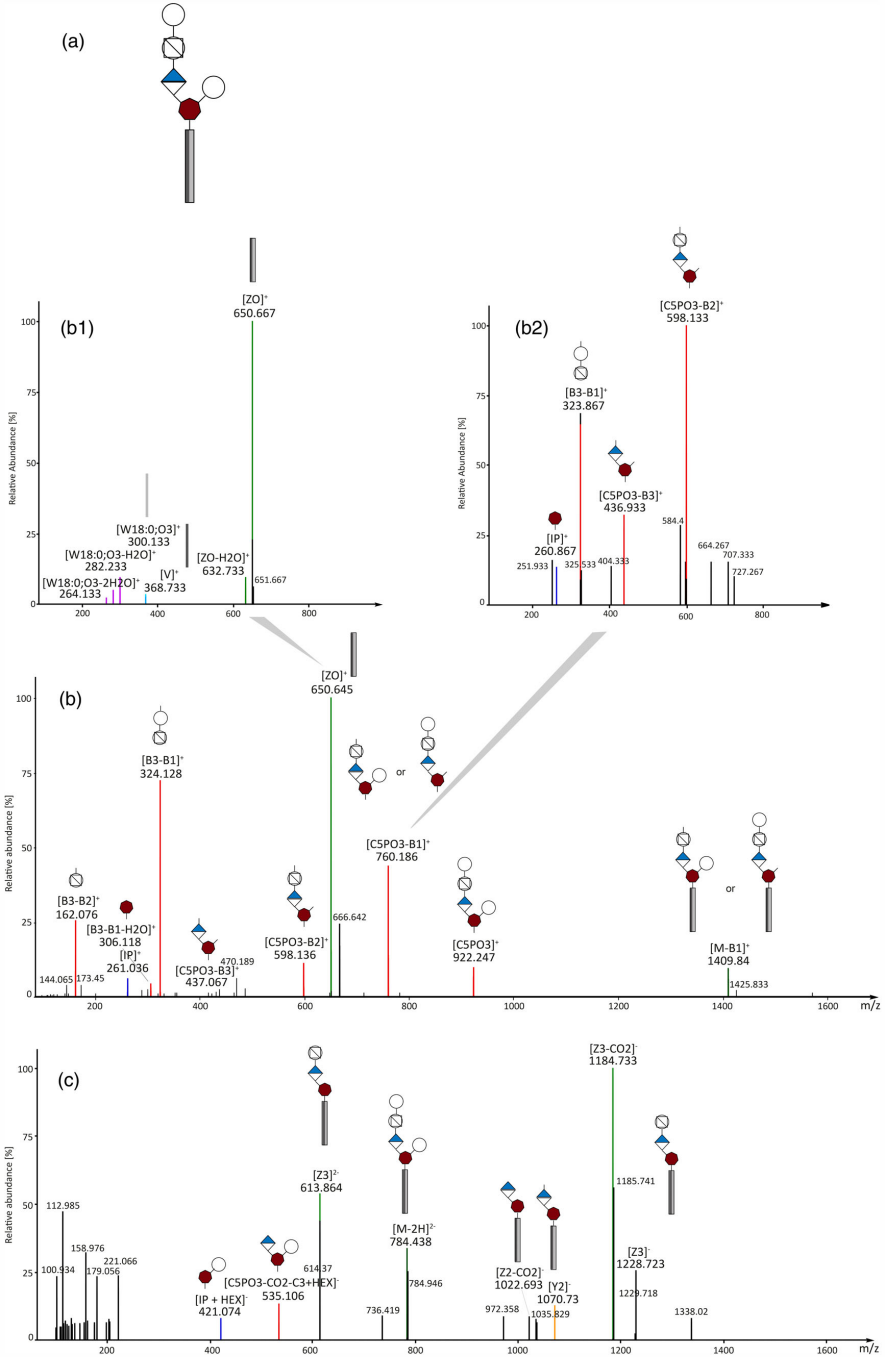
MS^n^ spectra of GIPC C‐HEX‐NH‐18:0;O3/22:0;O identified in barley samples. (a) Chemical structure of the compound. MS^n^ peaks are assigned to their fragments depicted in symbol nomenclature where the gray rectangles represent the fatty acid and sphingoid base while the sugar part is depicted as introduced in Figure 1. Fragments are named with letters (B, C, Y, Z, V, W) as introduced by Domon and Costello (1988). (b1) MS3 spectrum of the Z0^+^ fragment (650 *m/z*) in positive ion mode. (b2) MS3 spectrum of the C5PO3‐B1^+^ fragment (760 *m/z*) in positive ion mode. (c) MS2 spectrum of the [M‐2H]^2−^ precursor ion. The IP + HEX and C5PO3 – CO2 + HEX fragments indicate a branched GIPC structure and were detected in negative ion mode.

An exemplary structure of an annotated C‐series GIPC (C‐HEX‐NH‐18:0;O3/24:0) with 5 hexoses is depicted in Figure 5. MS2 spectra of C‐series GIPC show many diagnostic fragments of the polar sugar head group in positive ionization mode (Figure 5b) including the IP fragment at 261 *m/z*. The composition of the ceramide moiety can be determined by the positive MS3 spectrum of [Z0]^+^. In the spectrum, the W fragments correspond to the SPB, accompanied by two water loss fragments (W‐H_2_O, W‐2H_2_O; Figure 5c) and the V fragments correspond to the FA. From this example, it is clear that MS3 fragmentation is required for confirming the structure on the molecular species level, as V & W fragments are hardly ever present in MS2 spectra, and if so, then at low intensities. In the MS3 spectrum of [Z0]^+^, molecular species‐specific fragments are of high abundance. Further confirmation of the C‐series GIPC structure is provided by the MS3 spectrum of [C5PO3‐B1]^+^ (see Figure 5b2), which shows diagnostic B and C sugar head group fragments. Negative ion mode MS2 spectra of C‐series GIPC (Figure 5c) also show the branching fragment at 535 *m/z*.

In total, we identified 102 GIPC in the barley grain samples using the described method. 25 (25%) of the identified GIPC belong to series A with an NH2 group (HexN‐HexA‐IPC), 13 (13%) to series A with an acetyl group (HexNAc‐HexA‐IPC), 12 (12%) to series B with an NAc group (Hex‐HexNAc‐HexA‐IPC), 35 (34%) to series B with an NH2 group (Hex‐HexN‐HexA‐IPC), 11 (11%) to series C (Hex‐Hex‐HexN‐HexA‐IPC), and 6 (6%) to series D (Pen‐Hex‐Hex‐HexN‐HexA‐IPC). A complete list of all annotated GIPC and peak areas is provided in Tables S2 and S3. Across all samples, regardless of treatment or developmental stage, B‐series GIPC containing NH2 groups (B‐NH) comprised the highest relative share, followed by B‐series with acetyl groups (B‐NAc) and A‐series with NH2 groups (A‐NH). In contrast, A‐NAc, C‐NH, and D‐NH GIPC constituted smaller proportions of the total GIPC pool. These results demonstrate the potential of MS fragmentation patterns for comprehensive structural characterization of barley GIPC, uncovering glycan series‐specific features and ceramide diversity, including variations in glycan and lipid length, branching, hydroxylation, and double bond numbers.

### GIPC are temporally rearranged during barley grain development

**Figure 6:**
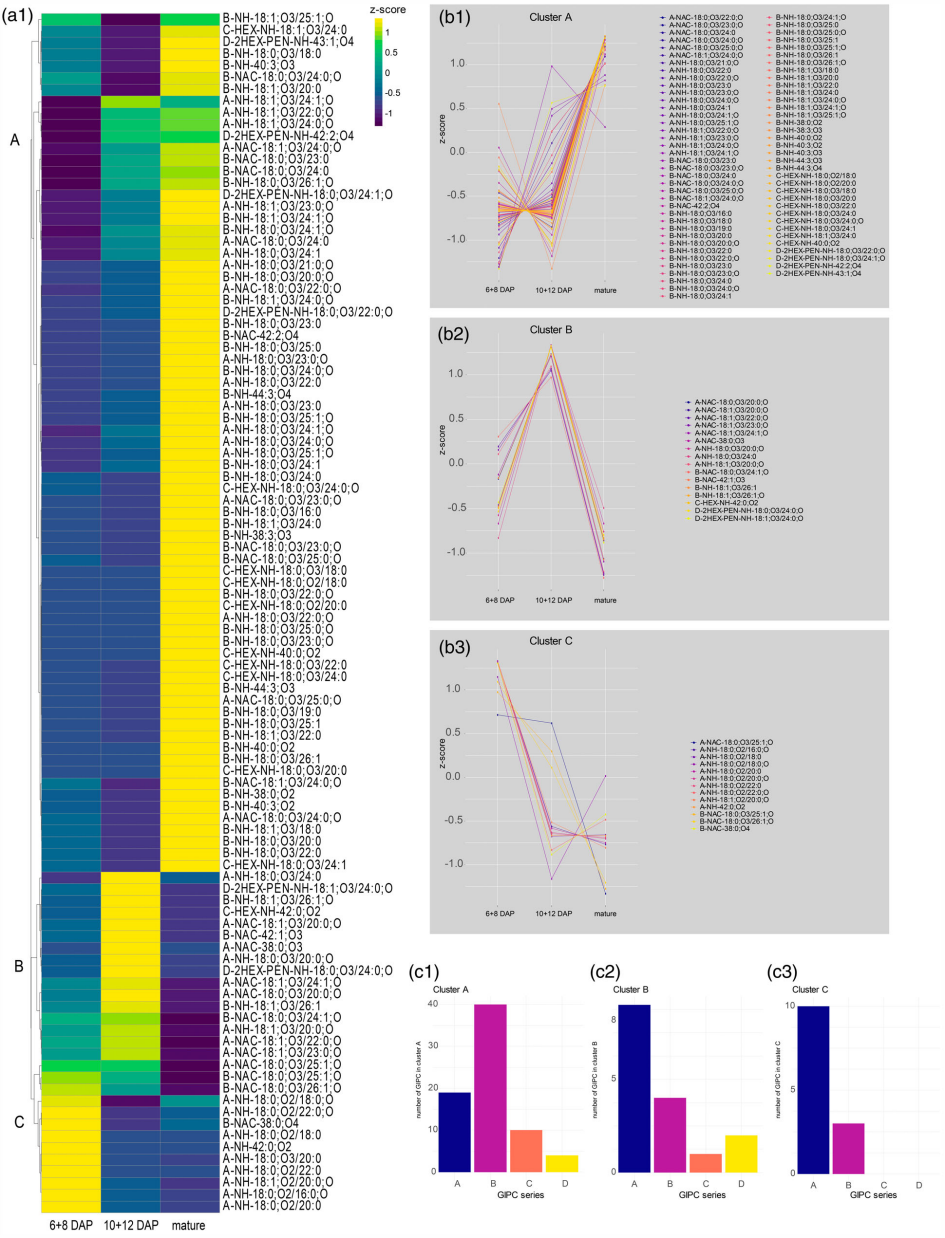
(a1) Hierarchical clustering analysis of GIPC during different barley grain development stages using correlation distance (Euclidean) plotted as a heatmap. (b1) z‐scores of cluster A GIPC species as indicated in the heatmap. (b2) z‐scores of cluster B GIPC species as indicated in the heatmap. (b3) z‐scores of cluster C GIPC species as indicated in the heatmap. (c1–c3) GIPC series affiliation of species in clusters A–C.

Since several physiological and morphological rearrangements are concomitant with barley grain development, we expected GIPC profile changes during this experiment. First, we analyzed the barley grain moisture reduction during grain development. GP grains undergo a massive moisture reduction from 50% at early (6 + 8 DAP), 18% at mid‐stage (10 + 12 DAP), and almost 0% at the mature stage (Table 1). Given the water loss, we applied our newly developed method to analyze the GIPC profile during grain development. Using RP‐HRMS^n^, we identified 102 distinct GIPC species. Principal component analysis (PCA) and hierarchical bi‐clustering analysis (HCA) revealed distinct patterns in the GIPC profiles. PC1, accounting for 40.3% of the variance, grouped samples in a stage‐specific manner, while PC2, representing 35.5% of the variance, successfully discriminated between early and mature stages (Figure S1). HCA identified three main clusters (Figure 6). Cluster A contained 73 GIPC, primarily characterized by B‐series GIPC (54.8%), which exhibited low abundance at the 6 + 8 DAP and 10 + 12 DAP stages compared to a marked increase at the mature stage. In cluster B, A‐series GIPC were most abundant (56.3%), followed by B‐series (25.0%) and D‐series (12.5%). Cluster C also showed A‐series GIPC to be prevailing (76.9%), while no C‐ & D‐series GIPC were identified in this cluster. The analysis revealed that during early grain development (6 + 8 DAP), A‐NH GIPC with a low degree of hydroxylations (SPB 18:0;O2 & 18:1;O2) were present at higher levels than other GIPC series. Additionally, clusters containing A‐NAc and B‐NAc were significantly upregulated at this stage. The three most abundant GIPC species, that is, B‐NH‐18:0;O3/24:0;O, B‐NH‐18:0;O3/24:0, and B‐NH‐38:0;O2 displayed higher concentrations in mature grains compared to earlier developmental stages. Most C‐series GIPC, specifically C‐HEX‐NH species with fatty acids (FAs) ranging from chain lengths of 18 to 24 carbons and with 2 to 4 hydroxylations, were also upregulated in mature barley grains. It is noteworthy that A‐NH GIPC with higher hydroxylations (4OH) as well as B‐NH GIPC with 4OH tended to be more abundant in mature grains, while the opposite was true for A‐NH species with 2OH. Furthermore, D‐series GIPC featured the longest glycan subunits, consisting of 7 sugar units, with a total of six D‐series GIPC identified in barley grains. Altogether, we observed more than 70% of all GIPC increasing during grain maturation, demonstrating significant GIPC rearrangement during grain development (Figure 6). Specifically, B‐series GIPC are most abundant at the mature stage, whereas A‐series GIPC decrease.

**Table 1:** Overview of the barley grain samples including the number of biological replicates (*n* = 3), and average fresh‐ and dry weights (g)

Growth stage (*n* = 3)	Fresh weight (g)	Dry weight (g)
6 + 8 DAP (heat stress)	0.12	0.05
6 + 8 DAP (control)	0.12	0.06
10 + 12 DAP (heat stress)	0.12	0.08
10 + 12 DAP (control)	0.11	0.09
Mature grains	0.10	0.10

### GIPC profile is affected during heat stress

**Figure 7:**
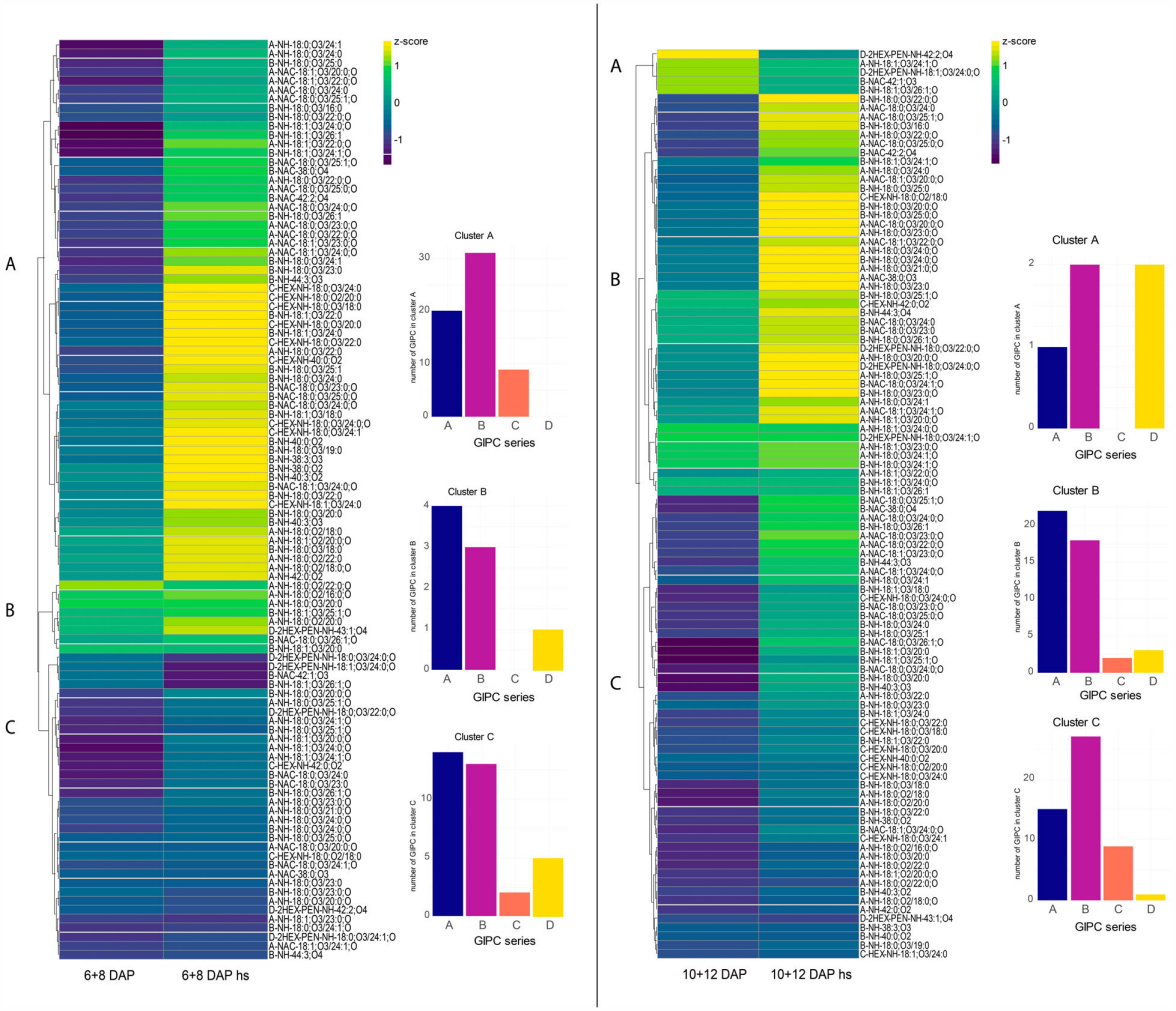
Hierarchical bi‐clustering analysis of GIPC in barley samples of different grain development stages and heat stress conditions. Euclidean correlation distance was used for clustering. 6 + 8 DAP = barley grains harvested 6 & 8 days after pollination (*n* = 3); 6 + 8 DAP hs = barley grains harvested 6 and 8 days after pollination and application of heat stress (*n* = 3); 10 + 12 DAP = barley grains harvested 10 and 12 days after pollination (*n* = 3); 10 + 12 DAP hs, barley grains harvested 10 and 12 days after pollination and application of heat stress (*n* = 3). Three clusters (A–C) are marked at the left side of the heatmap. The numbers of GIPC within each cluster and each lipid class are plotted as bar charts.

Next, we investigated whether heat stress influences GIPC profiles during barley grain development. Barley grains at developmental stages 6, 8, 10, and 12 DAP were exposed to 42°C for 4 h. To determine if this temperature affects the developing grain at the molecular level, we analyzed the levels of the 70‐kDa heat shock protein (HSP70), which is crucial in cellular processes and plays a key role after heat shock in plants (Aghaie & Tafreshi, 2020; Lee & Schöffl, 1996). Following the heat stress application, HSP70 abundance increased at all stages, particularly at the early stages of 6 and 8 DAP (Figure S2). Subsequently, we employed our newly developed method on the heat‐stressed grains (6 + 8 DAP, 10 + 12 DAP). At 6 + 8 DAP non‐stressed vs. heat‐stressed samples, three main clusters were identified in the HCA (Figure 7). 58% of all identified GIPC species are increased in heat‐stressed samples (cluster A), 8% are generally highly abundant (cluster B) and for 4% the abundance is only slightly affected when heat‐stressed (cluster C). The overall GIPC accumulation upon heat stress is in accordance with literature, which states that GIPC accumulation leads to desiccation tolerance in plants (Gasulla et al., 2013; Tarazona et al., 2015). A closer look into the clusters reveals that the GIPC profile varies over clusters A–C: while in cluster A, relatively high amounts of B‐, C‐, and A‐NAc‐series GIPC are present, A‐NH‐series and D‐series are predominantly present in cluster B and C. Overall, the abundance of B‐series GIPC is most induced by heat stress at 6 + 8 DAP, followed by series A. The HCA from 10 + 12 DAP heat‐stressed samples shows again three main clusters; however, more GIPC are induced compared to early development: cluster A, which contains 5% of the annotated GIPC, includes GIPC species that are downregulated in heat‐stressed samples in late development. Cluster B (43% of annotated GIPC species) shows GIPC species strongly increased under heat stress and is mainly composed of A‐series GIPC (49%). Cluster C shows a slighter increase in GIPC in heat‐stressed samples, especially B‐series. These data indicate that the overall GIPC accumulation induced by heat stress is dependent on the development stage of the grain. Especially B‐series GIPC species are upregulated at 6 + 8 DAP, whereas A‐series GIPC are strongly increased at 10 + 12 DAP.

## DISCUSSION

### Glycosphingolipidomics assay for highly glycosylated GIPC

Analytical methods for glycosphingolipids were traditionally designed to analyze either the glycan or the lipid moiety of these compounds, due to their substantial differences in physicochemical properties which can result in significant loss of structural information (Barrientos & Zhang, 2020). Consequently, intact GIPC analysis based on LC–MS/MS information was established (Markham et al., 2006). In this study, we introduce an LC–MS/MS glycolipidomics strategy allowing for a comprehensive analysis of complex multi‐glycosylated GIPC. Recently, the first RP‐HRMS assay for automated assignment of A‐series GIPC in various plant materials—including strawberries, raspberries, lettuce, and spinach—was introduced (Panzenboeck et al., 2020). Building on this strategy, we adapted the extraction protocol for barley grain analysis and expanded our assay to include annotation of GIPC A‐F series. Additionally, we incorporated fragments (IP + HEX and IP + HEX‐H_2_O) characteristic for branched glycan structures not previously reported in literature. As no commercial GIPC standards are available, C16‐lactosyl ceramide (LacCer 18:1;O2/16:0) was used as a chemically similar internal standard and added prior to GIPC extraction. Markham et al. demonstrated in 2006 that amphiphilic glycolipids require specialized lipid extraction strategies and that a mixture of hexane, water, and isopropanol (IPA) yields the highest amount of GIPC (Markham et al., 2006). To address the challenges of interfering glycerophospholipids, hydrolysis was proposed with methylamine/ethanol, potassium hydroxide (KOH) (Merrill. Jr et al., 2005), or sodium hydroxide (NaOH) (Blaas & Humpf, 2013). Alkaline hydrolysis of our barley samples enriched GIPC relative to bulk lipids, such as glycerophospholipids, and addressed the gelatinization issues associated with highly starch‐containing barley grains. The desludging process for less developed barley grains was challenging but improved by adding KOH during sample extraction. The final RP‐HRMS^n^ workflow achieved separation of GIPC within a 30 min chromatographic run and allowed in‐depth structural characterization through multistage fragmentation. The analytical figures of merit showed stable RP‐HRMS^n^ measurements (3–30% relative standard deviation (RSD) within a barley sample group after normalization) with consistent retention times (±0.1 min) in sequential positive and negative ion modes. Additionally, the same GIPC elution orders and major species were observed using the same reversed‐phase column on a different LC–MS system (Agilent Infinity 1290 UPLC coupled to a Sciex ZenoTOF 7600), demonstrating the reproducibility of the workflow. We implemented a novel GIPC annotation method based on decision rules that enables automated identification of complex A‐, B‐, C‐, and D‐series GIPC (R = OH, NH2, NAc, etc.), facilitated by the LDA software (Hartler et al., 2017). So far, most methods are based on targeted MRM mass spectrometry or combinations of GC–MS and LC–MS/MS, identifying only compounds specified in the method (Liu et al., 2021; Herrfurth et al., 2021). The group around Ivo Feussner has shown impressive results by MS‐based MRM methods for the combined analysis of lipids in glycosphingolipids to explore heat and drought stress as well as plant immunity (Aerts et al., 2024; Herrfurth et al., 2021; Tarazona et al., 2015). However, parallel lipids and glycosphingolipids analysis by MRM hampers the detection of higher GIPC series (e.g., Hex‐Hex‐Hex GIPC is primarily detected) as well as in‐depth structural information on the lipid and glycan part. The tailored extraction used in our study focuses on GIPC analysis while omitting other lipids using data‐dependent high‐resolution mass spectrometry and decision rule‐based data evaluation. The additional application of the ECN model and MS^n^ fragment search provides valuable information on yet unknown complex GIPC species in samples and the required high confidence in GIPC hits. Due to the absence of GIPC standards, the level of confidence that can be achieved for annotating GIPC still poses a central challenge. It is important to emphasize that no commercial GIPC standards are available, and the lack of clear criteria or standardized guidelines for validating GIPC hits increases the risk of structural over‐annotation. In order to enhance the degree of confidence in GIPC annotation in this work, we only included GIPC that met the following criteria: (1) MS2 spectra with characteristic fragments for the ceramide and sugar part in at least one ion mode were present; (2) they were detectable by accurate mass (5 ppm) in MS1 at the same retention time (±0.1 min) in both positive and negative ion modes; and (3) they satisfied the ECN model. In this work, the GIPC were annotated on the species level (sum composition, e.g., GIPC‐A‐OH‐40:1;O4) or the molecular species level (identified fatty acyl moiety and long chain base, e.g., GIPC‐A‐OH‐18:1;O3/22:0;O). As we could deduce the functional groups and either assigned species or molecular species level (Liebisch et al. 2013), the annotation level D was reached according to the classification system (the highest structurally resolved level is A, and the lowest is G) introduced by the metabolomics society (Rampler et al., 2021). MS3 spectra yielded fragments for distinguishing between isobaric compounds such as B‐NH 18:0;O3/24:1 and B‐NH 18:1;O3/24:0, which could be separated chromatographically (RT 17.70 and 18.54 min). A semiquantitative assessment of the GIPC changes was performed based on ratio comparisons to C16‐lactosyl ceramide (18:1;O2/16:0) and dry weight. If GIPC standards had been commercially available, actual absolute concentration measurements and GIPC quantification would have been possible. Furthermore, the incapability of standard LC–MS/MS workflows in resolving sugar stereochemistry remains unsolved and needs more research. Negative ion mode MS2 spectra of higher series GIPC provide useful information about sugar attachment order and branching (Buré et al., 2016). Using this workflow, we successfully annotated 102 GIPC species in barley grains seeds, consisting of 46 percent B‐series GIPC (35 B‐series Hex‐HexN‐HexA‐IPC and 12 Hex‐HexNAc‐HexA‐IPC). Cacas et al. (2013) also observed B‐series GIPC (with R = OH and R = N(Ac)) to be predominant in several other monocots (*Zostera noltii*, *Coix lacrymajobi*, *Allium porrum*, *Phalaenopsis* sp.). However, as indicated by Mamode Cassim et al. (2019), GIPC profiles are tissue‐specific and the analysis of seeds of several plants (*A. thaliana*, *Camelina sativa*, *Solanum lycopersicum* and the monocot *Oryza sativa*) revealed HexNAc‐HexA‐IPC A‐type GIPC as primary lipid class matching our GIPC profile observations.

### The abundance of B‐series GIPC is upregulated during grain development, including moisture loss

Given the decline of barley grain moisture from 50% (6 + 8 DAP) to 18% (10 + 12 DAP), eventually reaching nearly 0% at maturity (Table 1), our analysis of GIPC profiles during grain development revealed that A‐series GIPC are relatively decreasing from early development (6 + 8 DAP) towards the mature stage. In contrast, B‐series GIPC were mainly upregulated after 12 DAP and became the dominant series at maturity (Figure 6b1). Temporal regulation of GIPC was evident: B‐series GIPC mostly increased consistently throughout development, especially between 10 + 12 DAP and mature, whereas many A‐series GIPC exhibited an increase towards 10 + 12 DAP and decreased again towards the mature state or decreased consistently over grain development. The observed general GIPC accumulation in barley grains is consistent with studies linking GIPC to desiccation tolerance in plants (Gasulla et al., 2013; Tarazona et al., 2015). Specifically, the abundance of B‐series GIPC is mostly affected during grain development including moisture loss. However, the function of GIPC during grain development remains unknown.

### The abundance of B‐ and A‐series GIPC is temporally regulated in the barley grain at early‐ to mid‐stages after heat stress

**Figure 8:**
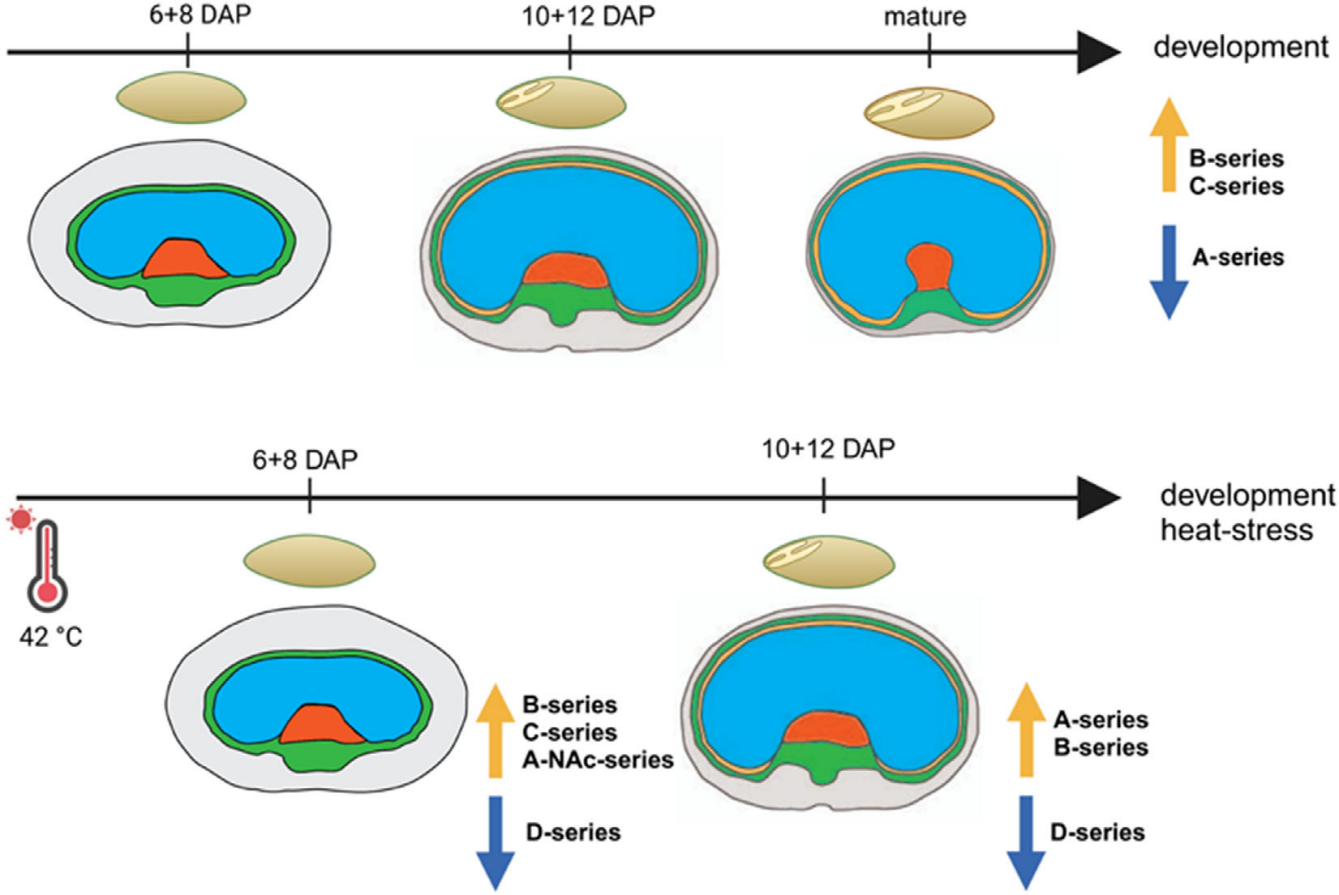
GIPC rearrangement during grain development and heat stress. Changing GIPC abundances are indicated with arrows. The pericarp is colored gray, the endosperm in blue, the embryo in red, the aleurone in beige, and the chlorenchyma in green. Created in https://BioRender.com.

Generally, heat stress application to barley grains induced a significant rearrangement and upregulation of specific series GIPC at early (6 + 8 DAP) and mid‐stage (10 + 12 DAP). Heat stress shifts the GIPC profile at early developmental stages (6 + 8 DAP) towards B‐series (NH and NAc), C‐series GIPC, and A‐NAc‐series GIPC (Figure 8). During heat stress application at 10 + 12 DAP, longer A‐NH‐species with high hydroxylation, as well as A‐NAc‐series GIPC, which generally have larger carbon chains, are upregulated. Interestingly, D‐series GIPC, such as D‐2Hex‐Pen‐NH‐18:0;O3/24:0;O, are downregulated under heat stress. Compared to the GIPC profile during grain development without stress application, B‐series GIPC are more abundant at 6 + 8 DAP heat stress and A‐series at 10 + 12 DAP heat stress, indicating a specific involvement of B‐ and A‐series GIPC in response to heat stress in barley grains.

### GIPC profile in the complex grain tissue—still a long way to go

Both experiments reveal temporal changes in GIPC profiles. However, the spatial regulation of GIPC is still unknown. Cereal grains represent complex morphological structures that consist of distinct layers including the hull, pericarp, testa, embryo, aleurone layer, and endosperm, each with unique spatiotemporal physiological functions and molecular mechanisms (Moore et al., 2016; Olsen, 2004). The outer coat serves as a protective barrier, enclosing both the embryo and the endosperm. The cereal endosperm comprises four major cell types: an epidermal layer of aleurone cells surrounding the starchy endosperm cells, a basal layer of transfer cells, and the cells of the embryo‐surrounding region (Olsen, 2001, 2004). The starchy endosperm thereby functions as a storage site, as it accumulates starch and seed storage proteins (Olsen, 2004). Grain elongation, primarily determined by the pericarp, ceases around 10 days after flowering (DAF; >10 days after pollination, DAP). After this point, grains thicken (<10 DAP), with the pericarp and endosperm being the predominant tissues early on. Beyond 10 DAF, the pericarp decreases in size, while the endosperm becomes the dominant tissue. At maturity, the pericarp is reduced to form part of the protective hull (Pielot et al., 2015), whereas the endosperm can account for up to 75% of the seed weight, with the persistent starchy inner cells dead and the aleurone layer secreting enzymes for germination (Young, 2000). Besides these morphological changes, the endomembrane system within the endosperm, which regulates the synthesis and transport of (seed) storage of proteins, is also affected, especially during early grain filling (6–12 DAP) (Ibl et al., 2014; Roustan et al., 2018, 2020; Shabrangy et al., 2018). Additionally, programmed cell death in the endosperm begins around mid‐stage (<12 DAP), marked by increased nuclease activity, DNA fragmentation, and loss of membrane integrity (van Doorn et al., 2011; Young, 2000), leaving the aleurone layer and embryo as the only viable tissues at maturity. Thus, the grain undergoes dramatic tissue rearrangements during grain development, and experimental results represent the current status of the grain stage. Considering these aspects, we present within this paper temporally regulated GIPC abundances. However, we did not specifically detect spatio‐regulation. To do that, laser‐microdissection microscopic analysis needs to be performed, which is beyond the focus of this work. Additionally, future proteomic and metabolomic analyses are necessary to unravel if the GIPC profile is changed due to less or more GIPC production or degradation.

### The different structure and diverse functions of B‐ and A‐series GIPC

We observed significant remodeling of GIPC during barley grain development and under heat stress. Using our RP‐HRMS^n^ workflow, we characterized GIPC fine structures in the glycan and ceramide moiety. The predominant GIPC types were B‐ and A‐series, but we also detected C‐ and D‐series, which consist of more saccharide units. Structural changes included variations in glycan length and ceramide fatty acid composition, with 18:0;O3 as the most prominent SPB. All GIPC contained 2–4 hydroxy groups which are crucial for stabilizing membranes and facilitating protein interactions through enhanced hydrogen bonding (Marquês et al., 2018). During the heat‐stressed later developmental stages (10 + 12 DAP), A‐series NH GIPC exhibited longer fatty acid chains and an overall increase in hydroxylation. These alterations in lipid length and hydroxylation are crucial for the plasma membrane structure and its interaction with the adjacent cell wall, enhancing membrane fluidity and stability under stress conditions like drought or high temperatures, which are essential for cell survival (Sharma et al., 2023).

An indispensable tool of poikilothermic organisms like plants is homeoviscous adaptation (HVA) in response to water loss and temperature changes, ensuring optimal fluidity as well as proper functioning and structural integrity of cell membranes (Cano‐Ramirez et al., 2021). Homeoviscosity in plants is largely regulated by the unsaturation of fatty acids in phosphatidylglycerol (PG) (Sakamoto et al., 2004) and to a lesser extent by sterols (Niu & Xiang, 2018). However, since GIPC has been suggested to play a role in facilitating homeoviscosity (Mamode Cassim et al., 2021), we cannot exclude that distinct GIPC may be involved in barley grains during HVA.

Notably, the structure of GIPC—especially the length—is crucial for binding the necrosis‐ and ethylene‐inducing‐like protein (NLP) toxin receptors (Lenarčič et al., 2017). This is further emphasized by the specific binding of NLP to A‐series GIPC, which may leave monocots like barley less susceptible to NLP toxins (Lenarčič et al., 2017). Our discovery that glycan branching was observed in B‐, C‐, and D‐series GIPC in all barley samples, but not in A‐series GIPC, suggests a potential role of branching in NLP binding and subsequently in susceptibility to pathogens. However, it is important to note that the specific function of the lack of branching in A‐series GIPCs remains unknown and warrants further exploration.

The presence of highly hydroxylated and branched GIPC at later developmental stages suggests their role in maintaining membrane integrity during grain maturation, a process marked by reduced moisture content and increased metabolic demands. These structural adaptations also play a role in pathogen resistance, especially under heat/abiotic stress conditions.

Our findings highlight the importance of in‐depth structural characterization of GIPC to understand its role in membrane stability and stress resilience during grain development. The presented glycosphingolipidomics assay provides structural information on glycan series, length, and branching, as well as lipid length, ceramide composition, and overall hydroxylation status. Further studies are needed to clarify its specific roles in membrane remodeling and stress response in distinct tissues within cereal grains.

## CONCLUSION

In this study, we present an innovative RP‐HRMS^n^ assay for comprehensive profiling of glycosyl inositol phospho ceramides (GIPC) in barley grains, addressing the limitations of traditional glycosphingolipid analysis methods. Combining accurate mass, retention time, MS^n^ fragmentation, and decision rule‐based criteria, the assay enabled high‐confidence annotation of 102 GIPC species, including novel branched glycan structures and sphingoid bases. The spatiotemporal regulation of GIPC during grain development and their remodeling under heat stress underscores their critical roles in membrane stability, stress adaptation, and pathogen resistance. Notably, B‐series GIPC with branched glycans and highly hydroxylated ceramide backbones dominates in later developmental stages in barley grain as it naturally dries, likely contributing to membrane stability and reduced pathogen susceptibility. Heat stress‐induced shifts in GIPC profiles further emphasize their potential role in desiccation tolerance and membrane protection under adverse conditions. The ability to resolve GIPC fine structures, such as glycan branching and ceramide hydroxylation, provides new insights into GIPC homeostasis and their biophysical roles in plant membranes.

The RP‐HRMS^n^ assay provides a significant advancement in automated GIPC analysis, offering a robust platform for investigating their functional diversity, localization, and biotechnological potential in crop stress resilience and pathogen defense. By resolving GIPC fine structures, such as glycan branching and ceramide hydroxylation, this open‐access, decision rule‐based workflow provides a valuable resource for the research community. With further development, including GIPC standards, this assay holds promise as a powerful tool for advancing plant lipidomics and developing crops with enhanced stress tolerance and resilience under adverse environmental conditions.

## EXPERIMENTAL PROCEDURES

### Material

Spring barley (*Hordeum vulgare*, L.) wild‐type variety Golden Promise plants were cultivated at the Department of Functional and Evolutionary Ecology (University of Vienna, Austria). All chemicals were of LC–MS grade. Formic acid and *n*‐hexane were bought from VWR (Vienna, Austria). Methanol (MeOH), acetonitrile (ACN), 2‐propanol (IPA), and water were purchased from Honeywell (Offenbach, Germany). Potassium hydroxide (KOH) was purchased from Carl Roth (Karlsruhe, Germany). Butylated hydroxytoluene (BHT) and ammonium formate (AF) were bought from Sigma‐Aldrich (Vienna, Austria). Complete protease inhibitor cocktail tablets were purchased from Roche (Basel, Switzerland). C16‐lactosyl(*ß*) ceramide (18:1;O2/16:0) (d‐lactosyl‐ß‐1,1′ N‐palmitoyl‐d‐erythro‐sphingosine) was bought from Avanti Polar Lipids, Inc. (Alabaster, Alabama, USA).

### Growth conditions and heat stress application

Wild‐type barley (*H. vulgare*, L.) cultivar Golden Promise (GP) was germinated in 10 cm^3^ pods (4 grains per pod) filled with soil. Plants were grown in growth chambers (Conviron Adapsis CMP 6010, Controlled Environment limited) starting with day/night cycles of 14/12°C, 70% humidity, and 12 h of light from 6 am to 6 pm during germination until 1–2 months after germination. As soon as the awns appeared, before pollination and anthesis, plants were moved to a growth chamber with 18/16°C day/night cycles, 70% humidity, and 14 h of light from 6 am to 10 pm. For pollination, plants were put in a greenhouse at 20–25°C/16–18°C day/night cycles and 70% humidity and were watered once a day. Once the plants began to dry and turn yellow after grain filling, they were not watered anymore, and the grains were harvested at maturity.

Plants at development stages 6 + 8 and 10 + 12 days after pollination (DAP) were exposed to heat stress experiments. The pollination (0 DAP) of the barley plants occurs at a point where awns are approximately 1 cm high. About 12 h before the heat gradient was applied, upon pollination, the plants were moved into a growth chamber (22°C, 70% humidity). A temperature gradient of 1°C increase every 20 min up to 42°C was applied to the plants. The peak temperature was kept for 4 h before the grains of the heat stressed plants were harvested directly thereafter. The conditions in the growth chamber were monitored using a universal serial bus (USB) temperature and humidity sensor (EasyLog‐USB). The control grains were collected 12 h before the heat gradient was applied to the test grains. After harvesting, grains were uncoated, immediately frozen in liquid nitrogen, and stored at −80°C.

### Sample preparation

Frozen barley grains were ground and homogenized with a ball mill with a frequency of 30 sec^−1^ for 1 min and 30 sec (Retsch MM 400, Dusseldorf, Germany). The metal attachment of the ball mill was previously cooled with liquid nitrogen. Plant material was weighed into glass vials (10 mL) using a CPA225D balance (Sartorius, Vienna, Austria). The following samples were used for heat‐stress experiments. Grains of barley plants collected 6 and 8 DAP were combined and homogenized with the ball mill. Grains harvested 10 and 12 DAP were also combined and homogenized together. Samples were taken before and after heat stress application in every developmental stage. Another sample was obtained from dry mature grains (Table 1). Approximately 100 mg dry weight plant material of each sample was weighed into glass vials.

### One‐phase extraction combined with alkaline hydrolysis

A solvent mixture of isopropanol (IPA), *n*‐hexane, and water was used according to the one‐phase extraction method for GIPC previously reported by Markham et al. (2013) and Panzenboeck et al. (2020). Blank extractions without plant material were performed using the same procedure. One protease inhibitor cocktail was dissolved in 50 mL water. 645 μL of protease inhibitor cocktail tablet solution in water and 1 mL of an approximately 0.01% BHT solution in IPA were added and vortexed. Subsequently, 15 μL of 100 μM C16‐lactosyl(*ß*) ceramide in IPA were spiked as an internal standard into all samples and vortexed. 1.155 mL IPA and 0.2 mL *n*‐hexane were added and the solution was vortexed again. The following incubation step was performed at 30°C for 15 min under constant shaking. Samples were centrifuged at 4°C for 10 min at 1000 rpm, and supernatants were transferred into new glass vials. The extraction step was repeated by adding 0.5 mL 0.01% BHT solution in IPA, 0.58 mL IPA, 0.1 mL *n*‐hexane, and 0.32 mL water to the pellet. Samples were vortexed and incubated for 15 min at 30°C under constant shaking, followed by centrifugation at 4°C for 10 min at 1000 rpm. Supernatants were combined and dried under a stream of nitrogen. Alkaline hydrolysis was performed by adding 1 mL 0.1 M KOH in methanol to the dried residues. After intense shaking and vortexing, samples were incubated for 2 h at 37°C. The pH was adjusted to 6–7 by adding concentrated formic acid. All samples were dried under a stream of nitrogen. Dried residues were reconstituted in 1 mL IPA:H_2_O (65:35). All samples were vortexed before and after ultrasonication at 30°C for 15 min. ClariStep filters (Sartorius, Vienna, Austria) were used to filter 0.5 mL of the sample solutions directly into HPLC vials.

### Reversed‐phase chromatography

Liquid chromatography was performed on a Vanquish UHPLC system (Thermo Fisher Scientific, Germering, Germany) using a C18 reversed phase Acquity ULPC HSS T3 column (2.1 mm × 150 mm, 100 Å, 1.8 μm, Waters, Vienna, Austria) equipped with a VanGuard Pre‐column (2.1 mm × 50 mm, 100 Å, 1.8 μm, Waters, Vienna, Austria). Chromatographic elution was performed at a column temperature of 40°C and a constant flow rate of 0.25 mL min^−1^ using a binary solvent system consisting of solvent A ACN:H_2_O (3:2; v/v) and solvent B of IPA:ACN (9:1 v/v). 0.1% formic acid and 10 mM ammonium formate were added to both solvents. Gradient elution was carried out as follows: 0–2 min 30% B, 2–3 min ramp to 55% B, 3–17 min ramp to 67% B, 17–22 min ramp to 100% B, 22–26 min 100% B. After each run, the column was equilibrated using 30% B for 4 min. Injection volumes of 10 μL were injected with an autosampler.

### High‐resolution mass spectrometry and multistage fragmentation

The UHPLC system was coupled to an Orbitrap‐IDX tribrid mass spectrometer (Thermo Fisher Scientific, Bremen, Germany) attached to a heated electro spray ionization (HESI) ion source. Detailed information about method settings and applied filters is shown in Method S1. Gradient duration was 30 min for each measurement, but ions were only detected from 2 to 30 min using a spray voltage of 3.5 kV in positive mode and 2.9 kV in negative mode. Ion transfer tube temperature was set to 275°C in positive mode and 300°C in negative mode. The following settings were applied in both ionization modes: sheath gas 40, auxiliary gas 8, and sweep gas 1. MS1 spectra were acquired by the Orbitrap with a resolution of 120 000, a scan range of 500–2000 *m/z*, and an S‐lens RF level of 50% in both ion modes. An automatic gain control (AGC) target in MS1 of 400 000 was applied. The minimum intensity threshold was set to 20 000 in positive mode. In negative ion mode, the intensity minimum was set to 10 000. MS2 spectra were acquired based on an inclusion list containing *m/z* ratios of singly and doubly charged GIPC belonging to series 0‐F. MS2 spectra were acquired using a higher‐energy collisional dissociation (HCD) with normalized collision energies of 23 in positive mode and 35 in negative mode, an MS2 AGC target of 50 000, and a *m/z* range of 300–1000. The Orbitrap resolution was set to 30 000 for MS2 spectra. In negative ion mode, the filter product ion trigger was used to generate MS3 spectra from fragment ions, which occur in MS2 spectra containing the typical fragments of the phospho inositol group [IP]^−^ (259 *m/z*) and [IP‐H_2_O]^−^ (241 *m/z*). MS3 spectra were acquired using collisional‐induced dissociation (CID) with a collision energy of 35 in both ion modes. The ion trap was used to detect the MS3 fragments with a rapid scan rate. MS3 AGC target was set to 10 000.

### Data analysis

The software Lipid Data Analyzer (version 2.9.0) was used for automated GIPC annotation and extraction of *m/z* values of identified GIPC (Hartler et al., 2017, 2020; Panzenboeck et al., 2020). All LDA parameters, settings, and an example of decision rules are provided in Method S2. The mass lists (see Table S1) including the *m/z* ratios of GIPC in negative and positive ionization mode were calculated with enviPat Web (Loos et al., 2015). Decision rules for GIPC series A, B, C, D, E, and F, applied for MS2 and MS3 spectra, were generated based on fragmentation pathways reported in the literature and adapted accordingly. To prevent false positive annotations, LDA results and MS^n^ spectra were manually inspected. MS1‐based relative quantification was carried out using the software Skyline (MacLean et al., 2010; Pino et al., 2020). Total peak areas were normalized to the respective dry weights and peak areas of the internal standard, and the average over the three biological replicates was taken or set to 0 if only one replicate was non‐zero. Principal component analysis was performed using MetaboAnalyst (Pang et al., 2024; Xia et al., 2009). To compare the profiles of GIPC species across samples, we applied row‐wise z‐score scaling and plotted the normalized data as heatmaps using the “pheatmap” R package (Kolde, 2010). The raw data (.mzml), inclusion lists, the LDA settings, as well as the LDA fragmentation rules, are available under ftp://massive.ucsd.edu/v09/MSV000097136/. The LDA version applied within this work can be downloaded here: https://genome.tugraz.at/lda2/lda_download.shtml.

### Sample preparation and western blot

For protein extraction, uncoated grains (3–4 per condition) were homogenized using a mortar and pestle under liquid nitrogen until receiving a whitish‐green powder which was either stored at −80°C or transferred into a tube to be further processed. 150 μL of phosphate‐buffered saline (PBS) extraction buffer (0.14 M NaCl, 1.4 mM Na_2_HPO_4_, 10.6 mM KH_2_PO_4_, adjusted to pH = 6 with HCl) was added to the ground grains and mixed. The suspension was incubated at 4°C while shaking at 400 rpm for 1 h. After that, the samples were centrifuged (4°C; 20 000 ***g***) for 3 min to separate proteins in solution from the cell debris in the pellet. The supernatant was either transferred into a fresh tube and stored at −80°C or further processed. The sample protein concentration was determined using a Bradford assay with a BSA (Bovine Serum Albumin) dilution series (0–10 mg/mL) as a standard. Western blots were performed as previously described (Roustan et al., 2020). The membrane was incubated with the 1st antibody (Anti HSP70, Agrisera, ASo8 371) at a ratio of 1:5000 in tris‐buffered saline with Tween20 (TBST) [10x TBST, 0.5 M Tris, 1.5 M NaCl, pH 7.5 (HCl)], either for 1 h at room temperature or overnight at 4°C after 30 min of incubation at room temperature. For the 2nd antibody, an Amersham ECL Rabbit IgG HRP‐linked whole antibody (from donkey; 1:50 000) was used and incubated for 1 h. The detection of the Western blot was performed using a GE Healthcare Amersham™ ECL Select™ western blotting detection reagent kit. For each membrane, 0.5 mL of solutions A and B were mixed, pipetted onto the membrane, and incubated for 3–5 min in the dark. The chemiluminescent signals were detected and after that, the membrane was incubated with Ponceau for 20–30 min and de‐stained with ddH_2_O. Alternative staining was performed with Coomassie Blue solution for 5 sec and de‐staining with an acetic acid‐isopropanol solution. The scans of the films and membranes were analyzed and quantified using ImageJ and Microsoft Excel. The background was removed by narrowing the intensity pattern, and the relative signal intensity traced by marking each intensity section on the histogram. The results indicated the intensity of the band as a numerical value.

## AUTHOR CONTRIBUTIONS

The RP‐HRMSn methodology was implemented by NT, MP, and ER. The formal barley analysis, including sample preparation, data acquisition and data evaluation, were performed by MP, NT and MS. Figures and tables for data visualization were prepared by MP and supported by ER, VI and AT. Analytical instrumentation, lab resources and student supervision were provided by ER and VI. MP and NT performed the LDA data analysis, with automated annotation and GIPC rule implementation supported by LML and JH. Funding acquisition, conceptualization, and project management were performed by ER and VI Conceptualization was also supported by LP and AT. The original draft writing was performed by MP, ER, VI and MS, followed by manuscript revision and editing by all authors. All authors have given approval to the final version of the manuscript.

## CONFLICT OF INTEREST

The authors declare no conflict of interest.

## Supporting information


**Figure S1.** Principal component analysis (PCA).

**Figure S2.** 70‐kDa heat shock protein (HSP70) abundances of samples 6–12 DAP and after heat stress application. (a) Western blots showing HSP70 protein‐coupled antibody signal of 6, 8, 10, and 12 DAP barley grains. Protein extracts of control and heat‐treated grains were loaded. In the first three lanes, protein extraction of unstressed control grains of respective grain stage of 100, 150, and 200% total protein content were loaded. The last lane contains equal total protein content as lane one (100%) of a heat‐stressed (42°C) grain of respective age stage. (b) Relative HSP70 signal intensities (y‐axis) of western blots from (A) of different barley grain stages (x‐axis) were analyzed using ImageJ. Loadings of 100, 150, and 200% of total protein content of unstressed control grains are compared to heat‐treated grains (H42°C).

**Table S1.** Mass lists of A–F‐series GIPC as used in the LDA.

**Table S2.** Detected GIPC including peak areas, retention times, and normalized area ratios of each molecular species.

**Table S3.** Annotated MS2 and MS3 fragments for each identified GIPC.

**Method S1.** Settings of the HRMS^3^‐methods including applied filters.

**Method S2.** An example of decision rules applied in the LDA.

## Data Availability

The data that support the findings of this study are openly available in MassIVE at ftp://massive.ucsd.edu/v09/MSV000097136/.
