# Supplementary figures and images for: Automated mass spectrometry‐based profiling of multi‐glycosylated glycosyl inositol phospho ceramides (GIPC) reveals specific series GIPC rearrangements during barley grain development and heat stress response
Source: Plant J. 2025 Jun 26;122(6):e70279. doi: 10.1111/tpj.70279 (PMC12201980; doi:10.1111/tpj.70279)

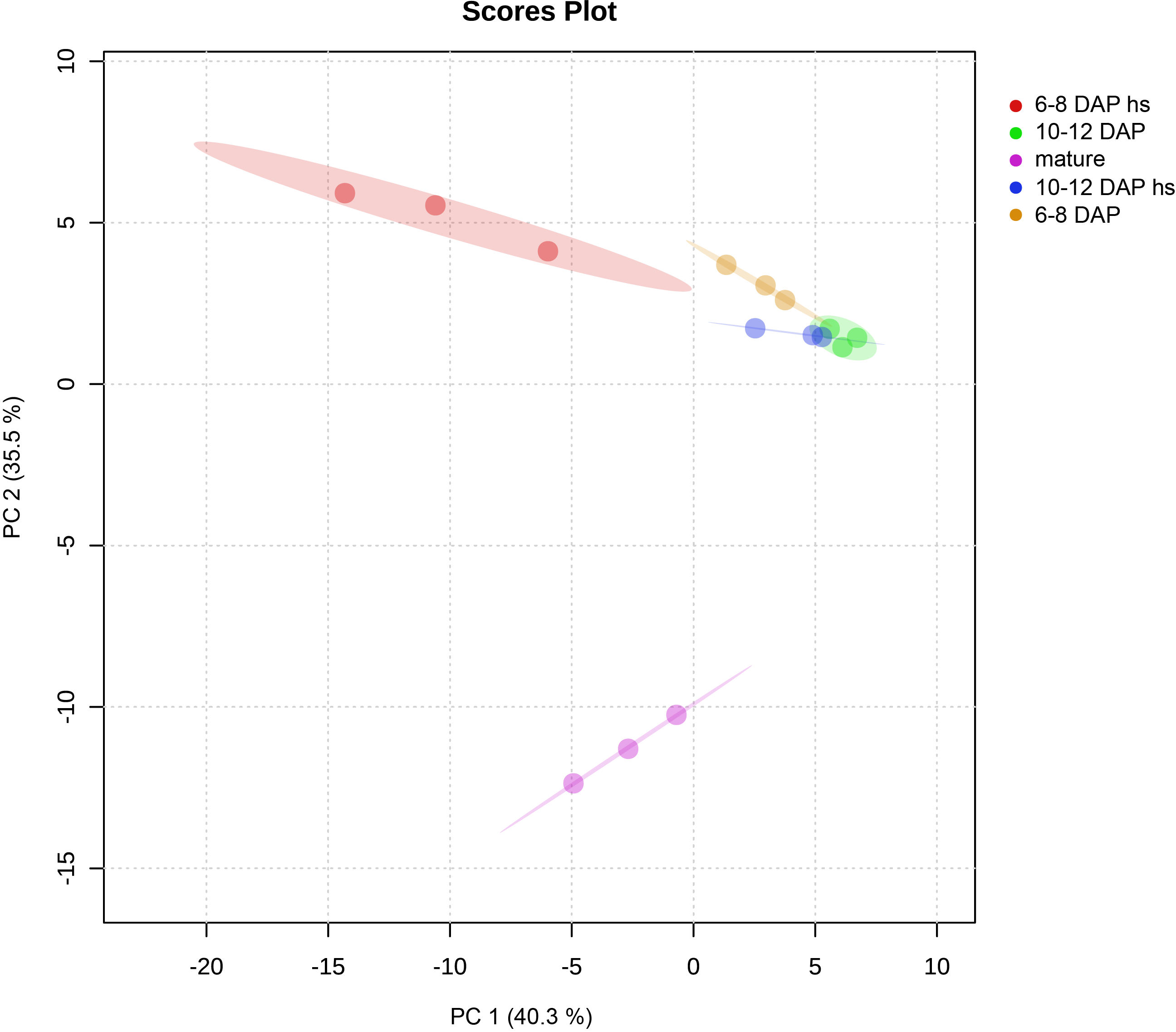

Supplement: Supplementary file 1 — Figure S1. Principal component analysis (PCA). [file TPJ-122-0-s006.png]

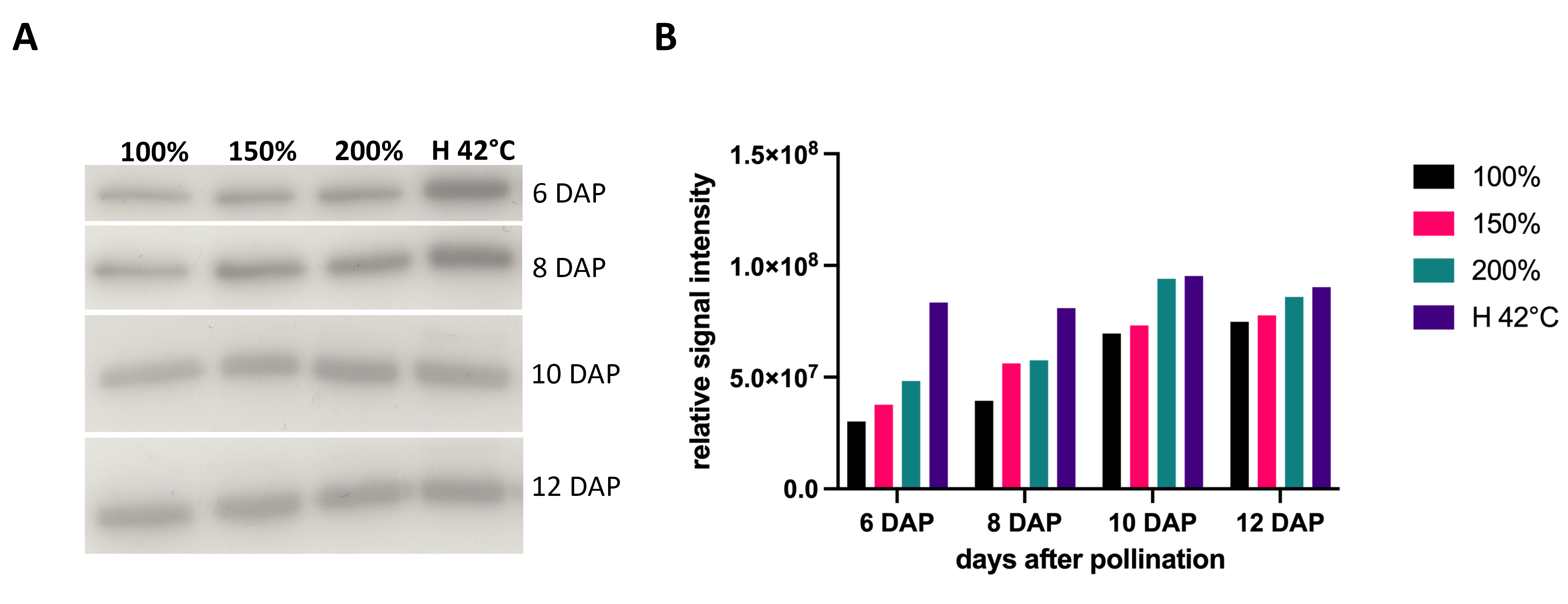

Supplement: Supplementary file 2 — Figure S2. 70‐kDa heat shock protein (HSP70) abundances of samples 6–12 DAP and after heat stress application. (a) Western blots showing HSP70 protein‐coupled antibody signal of 6, 8, 10, and 12 DAP barley grains. Protein extracts of control and heat‐treated grains were loaded. In the first three lanes, protein extraction of unstressed control grains of respective grain stage of 100, 150, and 200% total protein content were loaded. The last lane contains equal total protein content as lane one (100%) of a heat‐stressed (42°C) grain of respective age stage. (b) Relative HSP70 signal intensities (y‐axis) of western blots from (A) of different barley grain stages (x‐axis) were analyzed using ImageJ. Loadings of 100, 150, and 200% of total protein content of unstressed control grains are compared to heat‐treated grains (H42°C). [file TPJ-122-0-s004.png]
