# Supplementary material for: Automated mass spectrometry‐based profiling of multi‐glycosylated glycosyl inositol phospho ceramides (GIPC) reveals specific series GIPC rearrangements during barley grain development and heat stress response
Source: Plant J. 2025 Jun 26;122(6):e70279. doi: 10.1111/tpj.70279 (PMC12201980; doi:10.1111/tpj.70279)
Supplement: Supplementary file 6 — Method S1. Settings of the HRMS3‐methods including applied filters. [file TPJ-122-0-s003.docx]

Settings of the HRMS³-methods including applied filters.

| **Global Settings** | |
| --- | --- |
| Method Duration (min) | 30 |
| Spray Voltage | static |
| Spray Voltage Positive Ion (V) | 3500 |
| Spray Voltage Negative Ion (V) | 2900 |
| Gas Mode | static |
| Sheath Gas (Arb) | 40 |
| Auxiliary Gas (Arb) | 8 |
| Sweep Gas (Arb) | 1 |
| Ion Transfer Tube Temperature (°C) | pos : 275  neg: 300 |
| Vaporizer Temperature (°C) | 350 |
| Internal Mass Calibration | pos: Easy-IC  neg : Run Start Easy-IC |
| Application Mode | small molecule |
| Pressure Mode | standard |
| Default Charge State | 1 |
| Advanced Peak Determination | false |
| Xcalibur AcquireX enabled for method modifications | false |
| Mild Trapping | false |
| Start Time (min) | 2 |
| End Time (min) | 30 |
| Cycle time (sec) | 1 |
| **Master Scan** | |
| Desired minimum points across the peak | 6 |
| MS1 |  |
| Use Wide Quad Isolation | false |
| Detector Type | Orbitrap |
| MS1 Resolution | 120 000 |
| Mass Range | normal |
| Scan Range (*m/z*) | 500 – 2000 |
| Maximum Injection Time (ms) | 100 |
| MS1 AGC Target | 400 000 |
| Normalized AGC Target (%) | 100 |
| Microscans | 1 |
| Maximum Injection Time Type | custom |
| S lens RF level (%) | 50 |
| Use ETD Internal Calibration | pos: true  pos: false |
| DataType | profile |
| Source Fragmentation | false |
| Dynamic Exclusion | |
| Enhanced Resolution Mode | off |
| Exclude after n times | 1 |
| Exclusion Duration (s) | 5 |
| Mass Tolerance | ± 5 ppm |
| Use Common Settings | false |
| Exclude Isotopes | true |
| Perform dependent scan on single charge state per precursor only | false |
| Intensity Threshold | |
| Maximum Intensity | 1E+20 |
| Intensity Filter Type | Intensity Threshold |
| Minimum Intensity | pos: 20 000  neg: 10 000 |
| Relative Intensity Threshold | 0 |
| Do Data dependent if no targets are found | true |
| Targeted Inclusion Mass List | |
| Use Groups | false |
| Group ID must equal Isolation (m/z) | false |
| Set HCD Collision Energy per compound | false |
| Mass List Type | m/z |
| Mass Tolerance | ± 5 ppm |
| Ignore charge state requirement for unassigned ions | false |
| Include Intensity Threshold | false |
| Trigger Type | false |
| Prescursor Selection Range |  |
| Mass List | Inclusion List with double charged GIPC *m/z* |
| Precursor Selection Range | |
| Mass Range (*m/z*) | 500 – 2000 |
| Data Dependent Properties | |
| Data Dependent Mode | cycle time |
| Scan Event | 1 |
| **Scan ddMSnScan** | |
| Collision Energy Type | normalized |
| Desired minimum points across the peak | 6 |
| MS2 | |
| Isolation mode | Quadrupol |
| Isolation Offset | off |
| Isolation | false |
| Isolation Window | 1.5 |
| Reported Mass | original mass |
| Scan Range Mode | auto |
| Scan Priority | 1 |
| Activation Type | HCD |
| Collision Energy Mode | fixed |
| Collision Energy (%) | pos: 23  neg: 35 |
| Detector Type | Orbitrap |
| Orbitrap Resolution | 30 000 |
| Maximum Injection Time (ms) | 150 |
| AGC Target | 50 000 |
| Normalized AGC Target (%) | 100 |
| Inject ions for all available parallelizable time | false |
| Microscans | 1 |
| Maximum Injection Time Type | custom |
| Use ETD Internal Calibration | pos: true  neg: false |
| Data Type | profile |
| Source Fragmentation | false |
| Time Mode | unscheduled |
| Enhanced Resolution Mode | off |
| Maximum Intensity Threshold | |
| Maximum Intensity | 1E+20 |
| Intensity Filter Type | Intensity Threshold |
| Minimum Intensity | pos: 10 000  neg: 5 000 |
| Relative Intensity Threshold | 20 |
| Product Ion Trigger | |
| Use Groups | neg: false |
| Group ID must equal Isolation (m/z) | neg: false |
| Trigger only when at least n product ions from list are detected | neg: true |
| n | neg: 1 |
| Product ion(s) must be within top n | neg: false |
| Top N Product Ions | neg: 0 |
| Ignore charge state requirement for unassigned ions | neg: false |
| Mass list type | neg: *m/z* |
| Trigger only when correct Charge State of Product Ion is Detected | neg: true |
| Mass Tolerance | neg: ± 5 ppm |
| Product ion(s) must be above threshold (relative intensity, %) | neg: false |
| Product Ions Threshold | neg: 0 |
| Trigger Type | neg: false |
| Mass List Table (m/z) | -: 241.0113, 259.0219 |
| Precursor Selection Range MSn | |
| Mass Range | 300 – 1000 |
| Range relative to parent mass (%) | 0 – 1000 |
| Precursor Ion Exclusion | |
| Mass Tolerance | ± 5 ppm |
| Data Dependent Properties | |
| Number of Dependent Scans | 3 |
| **Scan ddMSnScan** | |
| Desired minimum points across the peak | 6 |
| MS3 | |
| Isolation Mode | Quadrupol |
| Isolation Offset | off |
| Isolation | false |
| Isolation Window | 1.5 |
| Reported Mass | original mass |
| MS2 Isolation Window (m/z) | 2 |
| Scan Range Mode | auto |
| Scan Priority | 1 |
| ActivationType | CID |
| Collision Energy Mode | fixed |
| Collision Energy (%) | 35 |
| Activation Time (ms) | 10 |
| Activation Q | 0.25 |
| Multistage Activation | false |
| Detector Type | Iontrap |
| Iontrap Scan Rate | rapid |
| Maximum Injection Time (ms) | 35 |
| AGC Target | 10 000 |
| Normalized AGC Target (%) | 100 |
| Inject ions for all available parallelizable time | false |
| Microscans | 1 |
| Maximum Injection Time Type | auto |
| Use ETD Internal Calbration | false |
| Data Type | profile |
| Source Fragmentation | false |
| Time Mode | unscheduled |
| Enhanced Resolution Mode | off |
