# Supplementary material for: Automated mass spectrometry‐based profiling of multi‐glycosylated glycosyl inositol phospho ceramides (GIPC) reveals specific series GIPC rearrangements during barley grain development and heat stress response
Source: Plant J. 2025 Jun 26;122(6):e70279. doi: 10.1111/tpj.70279 (PMC12201980; doi:10.1111/tpj.70279)
Supplement: Supplementary file 7 — Method S2. An example of decision rules applied in the LDA. [file TPJ-122-0-s002.docx]

Example of decision rules (B-NH series (H^+^ adduct)) for the Lipid Data Analyzer:

**GIPC_B_NH_H**

[GENERAL]

AmountOfChains=2

AmountOfLCBs=1

ChainLibrary=fattyAcidChains.xlsx

LCBLibrary=dLCB.xlsx

CAtomsFromName=\D*(\d+):\d+

DoubleBondsFromName=\D*\d+:(\d+)

BasePeakCutoff=0.05%

ChainCutoff=10%

SpectrumCoverage=10%

FaHydroxylationRange=0-1

LcbHydroxylationRange=2-3

RetentionTimePostprocessing=true

SingleChainIdentification=true

[HEAD]

!FRAGMENTS

Name=Precursor Formula=$PRECURSOR Charge=1 MSLevel=2 mandatory=false

Name=M-H2O Formula=$PRECURSOR-H2O Charge=1 MSLevel=2 mandatory=false

Name=M-B1 Formula=$PRECURSOR-C6H10O5 Charge=1 MSLevel=2 mandatory=false

Name=M-B2 Formula=$PRECURSOR-C12H21NO9 Charge=1 MSLevel=2 mandatory=false

Name=C4PO3_B2_437 Formula=C12H22O15P Charge=1 MSLevel=2 mandatory=false

Name=C4PO3 Formula=C24H41NO24P Charge=1 MSLevel=2 mandatory=false

Name=C4PO3-B1 Formula=C18H33NO19P Charge=1 MSLevel=2 mandatory=false

Name=Z0 Formula=$PRECURSOR-C24H42NO24P Charge=1 MSLevel=2 mandatory=false

Name=Z0mH2O Formula=$PRECURSOR-C24H44NO25P Charge=1 MSLevel=2 mandatory=false

Name=B1 Formula=C6H11O5 Charge=1 MSLevel=2 mandatory=false

Name=B1-B2 Formula=C6H12NO4 Charge=1 MSLevel=2 mandatory=false

Name=B2_NH Formula=C12H22NO9 Charge=1 MSLevel=2 mandatory=false

Name=IP Formula=C6H14O9P Charge=1 MSLevel=2 mandatory=false

Name=C4PO3_B2_MS3 Formula=C12H22O15P Charge=1 MSLevel=3 mandatory=false

Name=C4PO3_MS3 Formula=C24H41NO24P Charge=1 MSLevel=3 mandatory=false

Name=Z0_MS3 Formula=$PRECURSOR-C24H42NO24P Charge=1 MSLevel=3 mandatory=false

Name=Z0mH2O_MS3 Formula=$PRECURSOR-C24H44NO25P Charge=1 MSLevel=3 mandatory=false

Name=IP_MS3 Formula=C6H14O9P Charge=1 MSLevel=3 mandatory=false

[CHAINS]

!FRAGMENTS

Name=W Formula=$LCB-HO Charge=1 MSLevel=2 mandatory=false oh=1,2,3

Name=W-H2O Formula=$LCB-H3O2 Charge=1 MSLevel=2 mandatory=false oh=2,3

Name=W-2H2O Formula=$LCB-H5O3 Charge=1 MSLevel=2 mandatory=false oh=3

Name=W_MS3 Formula=$LCB-HO Charge=1 MSLevel=3 mandatory=false oh=1,2,3

Name=W-H2O_MS3 Formula=$LCB-H3O2 Charge=1 MSLevel=3 mandatory=false oh=2,3

Name=W-2H2O_MS3 Formula=$LCB-H5O3 Charge=1 MSLevel=3 mandatory=false oh=3

Name=V Formula=$CHAIN+H2N Charge=1 MSLevel=2 mandatory=false

Name=V_2 Formula=$CHAIN+HN Charge=1 MSLevel=2 mandatory=false

Name=V_MS3 Formula=$CHAIN+H2N Charge=1 MSLevel=3 mandatory=false

Name=V_MS3_2 Formula=$CHAIN+HN Charge=1 MSLevel=3 mandatory=false
